# Primary pigmented nodular adrenocortical disease associated with Carney complex: case report and literature review

**DOI:** 10.1590/S1516-31802006000600007

**Published:** 2006-11-01

**Authors:** Fabrícia Torres Gonçalves, Taciana Carla Maia Feibelmann, Cínthia Monteiro Mendes, Maria Luiza Mendonça Pereira Fernandes, Geraldo Henrique Gouvêa de Miranda, Agostinho Pinto Gouvêa, Paulo Tannús Jorge

**Keywords:** Cushing's syndrome, Hyperplasia, Adrenalectomy, Adrenal cortex diseases, Lentigo, Síndrome de Cushing, Hiperplasia, Adrenalectomia, Doenças do córtex suprarenal, Lentigo

## Abstract

**CONTEXT:**

Carney complex (CNC), a familial multiple neoplasm syndrome with dominant autosomal transmission, is characterized by tumors of the heart, skin, endocrine and peripheral nervous system, and also cutaneous lentiginosis. This is a rare syndrome and its main endocrine manifestation, primary pigmented nodular adrenal disease (PPNAD), is an uncommon cause of adrenocorticotropic hormone-independent Cushing's syndrome.

**CASE REPORT:**

We report the case of a 20-year-old patient with a history of weight gain, hirsutism, acne, secondary amenorrhea and facial lentiginosis. Following the diagnosing of CNC and PPNAD, the patient underwent laparoscopic bilateral adrenalectomy, and she evolved with decreasing hypercortisolism. Screening was also performed for other tumors related to this syndrome. The diagnostic criteria, screening and follow-up for patients and affected family members are discussed.

## RESUMO

**CONTEXTO:**

O complexo de Carney (CNC), uma síndrome de neoplasia múltipla familiar com transmissão autossômica dominante, caracteriza-se por tumores cardíacos, cutâneos, endócrinos e do sistema nervoso periférico, além de lentiginose cutânea.

**RELATO DE CASO:**

Devido à raridade da síndrome, bem como de sua principal manifestação endócrina, a doença adrenal nodular pigmentada primária (PPNAD), causa incomum de síndrome de Cushing ACTH-independente, relatamos o caso de uma paciente de 20 anos com história de ganho de peso, hirsutismo, acne, amenorréia secundária e lentiginose em face. Após estabelecido o diagnóstico de CNC e PPNAD, a paciente foi submetida a adrenalectomia bilateral via laparoscópica, evoluindo com melhora do hipercortisolismo. Também foi realizado rastreamento para os demais tumores relacionados à síndrome. Serão discutidos os critérios diagnósticos, o rastreamento e o acompanhamento dos pacientes e familiares afetados.

## INTRODUCTION

Carney complex (CNC) is a familial multiple neoplasia characterized by cardiac and cutaneous myxomas, multiple endocrine tumors (in the pituitary, thyroid, ovaries and testicles), primary pigmented nodular adrenocortical disease (PPNAD), breast and peripheral nervous system tumors and cutaneous lentiginosis syndrome. Up to 2001, only 338 cases had been reported.1 We describe a case in which the clinical manifestations were Cushing's syndrome due to PPNAD, and facial lentiginosis.

## CASE REPORT

**Figure 1:**
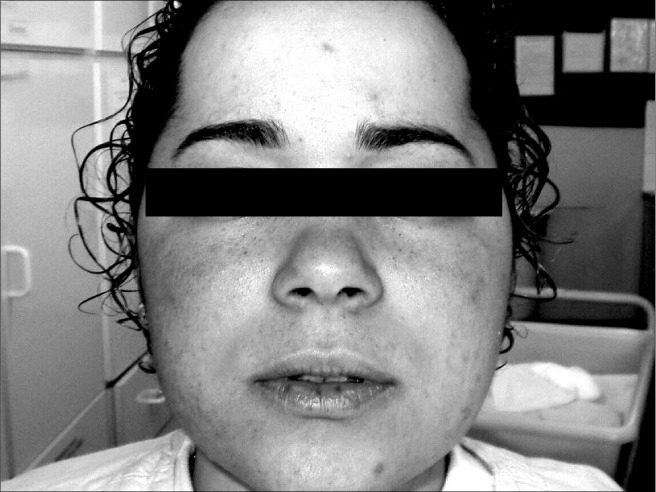
Lentigos on a 17-year-old patient with Carney complex.

A 17-year-old female was admitted to the Endocrinology Service of Hospital de Clínicas in Uberlândia, Minas Gerais, Brazil, with a history of weight gain, excessive hair, acne and secondary amenorrhea that had begun two years previously. The patient reported that she had started to have lentigos on her face at the age of 15 years (Figure 1). On examination, the patient presented cushingoid cheeks with discrete facial plethora and lentigos on the lips, infraorbital and conjunctive regions. Acne was found on her face, trunk and dorsal area and slight hirsutism (score 8 according to Ferriman & Gallwey^2^). The patient was overweight and body fat was concentrated in the trunk and abdomen. She also presented dorsal fat deposits. Her weight was 56.0 kg, her height was 1.48 and her body mass index (BMI) was 28 kg/m^2^. Her lung and heart auscultation were normal and she presented heart rate of 78 beats/min and arterial blood pressure of 130 x 80 mmHg. Her abdomen was painless, without any palpable masses, and presented purple stretch marks. Small ecchymoses were found on the lower limbs. She had a personal history of dyslipidemia and polycystic ovarian syndrome (PCOS), which had been diagnosed by a gynecologist who prescribed oral contraceptives. She said she had not made previous use of corticoids. In her family, her mother and a healthy brother had lentiginosis. The parents were not blood-related.

The main laboratory tests performed are described in Table 1. Other test results included: normal echocardiogram; normal mammography; normal pelvic ultrasound; and thyroid ultrasound showing the presence of diminutive thyroid bilateral cysts, of which the largest was 5.5 mm.

**Table 1:** Laboratory tests results for a 17-year-old girl with lentigo, acne, hirsutism and polycystic ovarian syndrome, who was overweight

Plasma cortisol^1^	8:00 = 29.4 μg/dl 16:00 = 32.7 μg/dl midnight serum cortisol = 33.9 μg/dl
UFC^1^ (in 24-hour urine collection)	1,685 μg/24h (92- 413 μg/24h)
Plasma cortisol1 after overnight 1 mg dexamethasone suppression test	8:00 = 32.3 μg/dl
Plasma cortisol^1^ after high-dose dexamethasone suppression test (0.5 mg every 6 hours for 48 hours)	15.3 μg/dl
Plasma ACTH1 (four samples)	08:00 = 3.1 pg/ml (< 46 pg/ml) 08:00 = 2.4 pg/ml (< 46 pg/ml) 00:00 = 1.4 pg/ml (< 46 pg/ml) 08:00 = 2.0 pg/ml (10 - 60 pg/ml)
Plasma cortisol1 after dexamethasone 2 mg (0.5 mg every 6 hours for 48 hours) followed by dexametha-sone 8 mg (Liddle test):	Urine free cortisol/24 hours: 263 μg (10 - 90 μg/24h) — baseline Urine free cortisol/24 hours after dexamethasone 2 mg for 48 hours: 279 μg Urine free cortisol/24 hours after dexamethasone 8 mg for 48 hours: 401 μg **(Paradoxical response = 52% increase in urinary free cortisol excretion)**
DHEAS	569 ng/ml (600 - 5,000 ng/ml)
Testosterone	575 pg/ml (80 - 970 pg/ml)
Thyroid hormones	TSH = 0.3 mIU/ml; free T4 = 1.1 ng/dl; thyroid autoantibodies (anti-thyroglobulin and anti-thyroperoxidase) were not detectable
Prolactin	5.0 ng/dl
GH (baseline sample) IGF-1	0.63 ng/ml (0.06 - 5.0 ng/ml) 346.0 ng/ml (116.0 to 358.0 ng/ml)
Other laboratory tests	Fasting serum glucose: 77.4 mg/dl Total cholesterol: 259 mg/dl LDL cholesterol: 155 mg/dl HDL cholesterol: 78.9 mg/dl Triglycerides: 130 mg/dl

*UFC = urinary free cortisol; 1 Measured by chemiluminescent immunoassay; ACTH = adrenocorticotropic hormone-independent; DHEAS = dehydroepiandrosterone sulfate; GH = growth hormone; IGF-1 = insulin-like growth factor 1; TSH = thyroid-stimulating hormone; LDL = low-density lipoprotein; HDL = high-density lipoprotein.*

**Figure 2:**
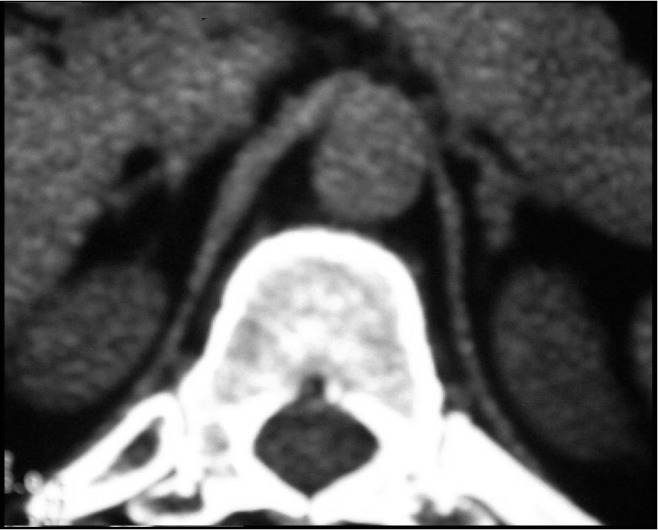
Adrenal computed tomography scan of the adrenals of a patient with Carney complex.

The patient was diagnosed as having adrenocorticotropic hormone-independent (ACTH-independent) Cushing's syndrome, and a computed tomography (CT) scan was performed to investigate the adrenals (Figure 2). The medical report described the adrenal glands as normal-sized with discrete irregularity of the left adrenal gland outline.

**Figure 3:**
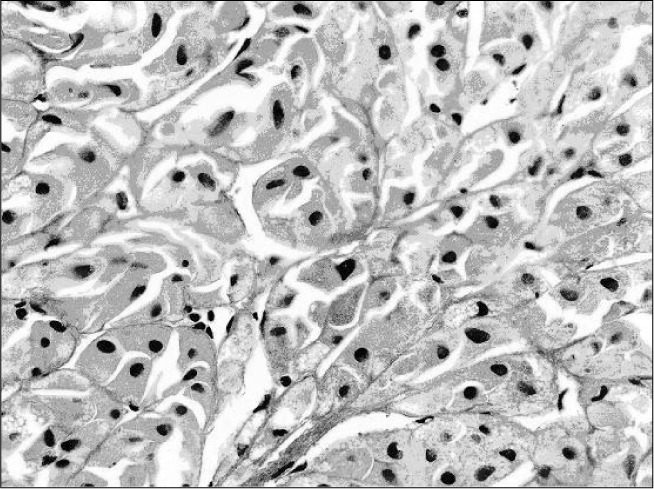
Photomicrograph of adrenal cortex (patient with Carney complex): eosinophil cells containing brownish pigment in their cytoplasm.

On the basis of the clinical and laboratory data, the main hypothesis was hypercortisolism due to PPNAD associated with Carney complex. The patient underwent laparoscopic left adrenalectomy and, about one month later, right adrenalectomy. Both procedures were performed under general anesthesia by means of a lateral transabdominal approach. No incidents occurred during the procedures and the patient was discharged on the fourth day after surgery. The diagnosis was then confirmed anatomopathologically: macroscopic examination of the external surface and sectioned surface revealed multiple nodules and some of the nodules were brownish-yellow. Histological sections under microscopy showed, mainly in the cortical gland, benign nodular and circumscribed proliferation of lipid-rich eosinophil cells similar to normal cells in the adrenal reticular zone. Many of these cells contained brownish pigment in their cytoplasm (Figure 3).

## DISCUSSION

The Carney complex (CNC) is currently considered to be a multiple neoplasia syndrome. It was first described by Carney et al. in 1985 and is characterized by pigmented lesions on the skin; cardiac and cutaneous myxomas; multiple endocrinal tumors (adrenal, testicular or ovarian, thyroid, and hypophysis); and, less frequently, psammomatous melanotic schwannoma, ductal adenoma of the breast and rare bone tumors.^1,3^

It is an autosomally dominant inherited syndrome. The clinical manifestations are very variable among patients, even in the same family.^4^ Approximately half of the families with CNC that have been studied presented mutations in the gene *PRKAR1A*, which is located on the long arm of chromosome 17 (17q22-24). This gene acts as a classical tumor suppressor, responsible for the production of the type 1α regulatory subunit of protein kinase (PKA). PKA is related to important pathways for endocrinal signaling. The R1α subunit inhibits PKA function, and *PRKAR1A* mutations originate a truncated protein that is functionally null, thus leading to increased intracellular signaling via PKA, and consequent endocrinal hyperactivity or tumor formation.^4-6^ In most other families, mutations of the 2p16 locus have been found, thus suggesting their involvement in the pathogenesis of the disease.^4,7,8^

The diagnosis of the syndrome is based on criteria proposed in 2001 by Stratakis et al.,^4^ from descriptions of a total of 338 patients around the world. Patients are considered to have CNC if two major criteria or one major criterion and two supplementary criteria are present, as shown in Table 2.

**Table 2:** Diagnostic criteria for Carney complex. In order to be diagnosed as a case of Carney complex, a patient must either: 1) exhibit two of the manifestations of the diseases listed, or 2) exhibit one of these manifestations and meet one of the supplemental criteria.^4^

1. Spotty skin pigmentation with typical distribution (lips, conjunctiva and inner or outer canthi, vaginal and penile mucosa)
2. Myxoma (cutaneous and mucosal)
3. Cardiac myxoma
4. Breast myxomatosis or fat-suppressed magnetic resonance imaging findings suggestive of this diagnosis
5. Primary pigmented nodular adrenal disease or paradoxical positive response of urinary glucocorticosteroids to dexamethasone administration during Liddle test
6. Acromegaly due to growth hormone-producing adenoma
7. Large cell calcifying Sertoli cell tumor or characteristic calcification on testicular ultrasonography
8. Thyroid carcinoma or multiple, hypoechoic nodules on thyroid ultrasonography, in a young patient
9. Psammomatous melanotic schwannoma
10. Blue nevus, epithelioid blue nevus (multiple)
11. Breast ductal adenoma (multiple)
12. Osteochondromyxoma
***Supplemental criteria:*** 1. Affected first-degree relative 2. Inactivating mutation of the *PRKAR1A* gene

In the present case, CNC was suspected because of the lentigos on the face, together with ACTH-independent Cushing's syndrome with normal adrenals, which suggested PPNAD. However, it is worth emphasizing that, in cases like this, because of the patient's low levels of ACTH and normal adrenal imaging, a hypothesis of exogenous glucocorticoid should also be persistently investigated and ruled out. Furthermore, it is important to make sure that the sampling and assaying of ACTH have been performed adequately: blood should be drawn into a frozen tube containing ethylenediamine tetraacetic acid (EDTA), followed by immediate centrifugation and cooling of the sample. Otherwise, the ACTH values may be falsely found to be low and thereby induce diagnostic error.^9^

PPNAD is a rare form of ACTH-independent Cushing's syndrome that may occur alone, but is found to be associated with CNC in 90% of the cases.^10^ It is the most common endocrinal hyperactivity in these patients, and has been described in 25% of them. This incidence, however, is probably an underestimate, because of untypical and subclinical cases, and the cycles of the disease. In studies on autopsies of patients with CNC, PPNAD was observed in almost all cases.^4,10,11^

Although our patient presented a classical picture of Cushing's syndrome, her two-year history of amenorrhea, which was thought to have been caused previously by PCOS, and her short stature could be indicative of a disease that emerged atypically a long time earlier. Prolonged exposure to undiagnosed hypercortisolism may also explain cases of peculiarly severe osteoporosis in patients with PPNAD.^7,11^

Paradoxical cortisol secretion responses after performing the Liddle test (two days of baseline collection, two days of dexamethasone 0.5 mg orally every six hours followed by two days of 2 mg orally every six hours and a new sample on the sixth day of the test) showed an increase of more than 50% in 24-hour urinary free cortisol (UFC) in relation to the baseline value. This is considered to be one of the major criteria for CNC.^10,12^ Stratakis et al. studied 16 cases of PPNAD and compared them with control patients with adrenal adenoma and macronodular adrenal hyperplasia.^12^ They found that an increase of 50% or more in 24-hour UFC made it possible to identify a great number of PPNAD cases (approximately 70%), but only a few adenoma cases and no macronodular hyperplasia cases presented the same type of response. On the other hand, a 100% increase in 24-hour UFC in relation to the baseline value identified PPNAD cases alone (100% specific). Moreover, Stratakis et al. showed that the same response pattern occurs in the asymptomatic cases, cyclical cases and atypical types of Cushing's syndrome that frequently occur in PPNAD. This is therefore useful for early diagnosis of CNC in patients suspected of this disease.^12^ In our case, a paradoxical response was found with a 52% increase in 24-hour UFC after performing the Liddle test.

Our patient underwent bilateral adrenalectomy. The main advantages of laparoscopy over the open procedure are shorter inpatient time, reduced blood loss and lower general incidence of complications. The histopathological findings from the surgical specimen were characteristic of primary pigmented nodular cortical and adrenal hyperplasia.

The brownish pigmented lesions (lentigos) noted on the lower lips, infraorbital region and conjunctive tissue, which according to the patient had emerged since she was around 15 years old, are typical for people with this syndrome and are present in approximately 77% of the cases. Normally they are spread out, intense and dense during puberty. However, other types of lesions such as blue nevi, black and brown blemishes and unpigmented lesions may be found.^4,7,11^

Even after fulfilling the major criteria (described above), screening for other components of the syndrome was performed. Although cardiac myxomas were not found, these should be screened annually by Doppler echography because of their seriousness and the fact that they may emerge at any moment during the course of the disease.^1,13,14^

The thyroid alterations found were compatible with lesions described in CNC cases: typically small, multiple, hypoechoic, solid cystic or mixed. They are present in 75% of patients and may correspond to simple cysts, adenomas or carcinomas. A new ultrasound should be performed only if necessary.^15^ Thyroid function in CNC patients is frequently normal. The finding of initially suppressed thyroid-stimulating hormone (TSH) in our patient, with normal FT4 and negative antibodies, was considered to be due to hypercortisolism, since it became normalized after bilateral adrenalectomy.

Mammography and pelvic ultrasound were performed to rule out ductal adenomas or breast and cyst myxomas, or ovarian tumors, respectively. As no lesions were detected, these tests should only be repeated if there is clinical suspicion, because of the low risk of malignity of these tumors.^1,16^

Pituitary tumors may be present, but they are not always clinically manifested. Increased prolactin and growth hormone (GH) occur in more than 75% of patients with CNC, which justifies routine measuring of these hormones. In our case, prolactin, GH and insulin-like growth factor 1 (IGF-1) were normal. Patient follow-up should be performed, measuring IGF-1 or GH during oral glucose tolerance test (GTT) annually.^1^

As CNC is an autosomal dominant inherited syndrome and has high penetrance (almost 100%), first-degree relatives should begin screening (clinical, laboratory and imaging tests, if necessary) to investigate any abnormalities in the complex.^4,7^ Screening for *PRKAR1A* mutations in those affected and their families is not recommended at this stage, since such mutations are only present in a little more than 40% of the families with CNC.^4,11^

## CONCLUSION

PPNAD should be suspected in cases of ACTH-independent Cushing's syndrome with normal adrenal imaging, and due care should always be taken to avoid the use of exogenous glucocorticoids by any route. Screening for Carney Complex and its complications, which are often fatal, should be undertaken in all cases of PPNAD, as well as screening for the syndrome in the patient's family members.
